# Disease management in two sympatric *Apterostigma* fungus‐growing ants for controlling the parasitic fungus *Escovopsis*

**DOI:** 10.1002/ece3.7379

**Published:** 2021-05-02

**Authors:** Yuliana Christopher, William T. Wcislo, Sergio Martínez‐Luis, William O.H. Hughes, Nicole M. Gerardo, Hermógenes Fernández‐Marín

**Affiliations:** ^1^ Centro de Biodiversidad y Descubrimiento de Drogas Instituto de Investigaciones Científicas y Servicios de Alta Tecnología (INDICASAT AIP) Clayton República de Panamá; ^2^ Department of Biotechnology Acharya Nagarjuna University Guntur India; ^3^ Smithsonian Tropical Research Institute Panama República de Panamá; ^4^ School of Life Sciences University of Sussex Brighton UK; ^5^ Department of Biology Emory University Atlanta GA USA

**Keywords:** Attini, coevolution, disease ecology, fungal symbiont, mycoparasite, parasitism

## Abstract

Antagonistic interactions between host and parasites are often embedded in networks of interacting species, in which hosts may be attacked by competing parasites species, and parasites may infect more than one host species. To better understand the evolution of host defenses and parasite counterdefenses in the context of a multihost, multiparasite system, we studied two sympatric species, of congeneric fungus‐growing ants (Attini) species and their symbiotic fungal cultivars, which are attacked by multiple morphotypes of parasitic fungi in the genus, *Escovopsis*. To assess whether closely related ant species and their cultured fungi are evolving defenses against the same or different parasitic strains, we characterized *Escovopsis* that were isolated from colonies of sympatric *Apterostigma dentigerum* and *A*. *pilosum*. We assessed in vitro and in vivo interactions of these parasites with their hosts. While the ant cultivars are parasitized by similar *Escovopsis* spp., the frequency of infection by these pathogens differs between the two ant species. The ability of the host fungi to suppress *Escovopsis* growth, as well as ant defensive responses toward the parasites, differs depending on the parasite strain and on the host ant species.

## Graphical abstract

We studied two sympatric Apterostigma fungus‐growing ants (Attini) species and their symbiotic fungal cultivars which are attacked by multiple morphotypes of parasitic fungi in the genus, Escovopsis. We assessed in vitro and in vivo interactions of these parasites with their hosts to understand, in the context of a multihost, multiparasite system, the evolution of host defenses and parasite counterdefenses. While the ant cultivars are parasitized by similar Escovopsis spp., the frequency of infection by these pathogens differs between the two ant species. The ability of the host fungi to suppress Escovopsis growth, as well as ant defensive responses toward the parasites, differs depending on the parasite strain and on the host ant species.

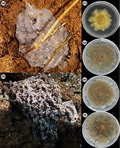

## 1 INTRODUCTION

Individuals within insect societies receive benefits and share responsibilities for the maintenance and propagation of their group (Hölldobler & Wilson, 1990; Weber, 1972). Some challenges require individual responses, and others require collective action (Hart, 1990). Adaptations at individual and social levels enable the development of task allocation strategies, including those for the prevention and control of disease (Schmid‐Hempel, 1995, 1998; Wilson, 1971). Variation in responses of individuals from different colonies could result in the development of different disease management strategies among colonies or species (Franceschi et al., 2005). Moreover, at local scales, when two or more species are challenged by the same pathogens, complementary defensive responses may reduce the prevalence or overall impact of the parasites by decreasing the likelihood that a pathogen evolves counterstrategies to a single host species.

When alternative host species are present, these hosts may function as a general pool of acceptable hosts with which a parasite evolves. To the extent that each host has unique defense strategies, it is potentially harder for a parasite to evolve to overcome all defenses. Cocktails of antimicrobial chemicals may be used to treat pathogens in order to minimize the likelihood of the evolution of resistance (Raymond, 2019). The fungus‐growing ant system provides an excellent opportunity to explore the degree to which different pathogens attack sympatric hosts, and how the hosts respond. This is in part because of the experimental tractability of the ants, the ants’ cultivated fungi, their common fungal parasites (*Escovopsis* spp.), and other associated microbes (Currie et al., 1999; Currie et al., 1999; Sen et al., 2009).

Previous work on *Escovopsis* infections in other sympatric, closely related attine species [i.e., *Cyphomyrmex costatus*, *C*. *muelleri,* and *C*. *longiscapus* (Birnbaum & Gerardo, 2016; Gerardo et al., 2004); *Atta* spp. and *Acromyrmex* spp. (Taerum et al., 2010)] indicates that colonies can be infected by the same parasites, though individual parasite strains may be more likely to infect colonies of one species than the others. In *Cyphomyrmex*, observed patterns of natural infections are consistent with differences in the parasites’ abilities to infect the ants’ gardens in experimental infections (Birnbaum & Gerardo, 2016; Gerardo et al., 2004). These differences, however, have not been clearly linked to ant behavioral defenses toward the parasites. Here, we test whether the colonies of two sympatric ant species, *Apterostigma dentigerum* and *A. pilosum*, are infected by the same parasite strains. We characterize the diversity of these parasites both genetically and phenotypically. We then assess whether the outcomes of parasite infections are similar in both colony types or whether these hosts present unique defensive challenges for these parasites to overcome.

## 2 MATERIALS AND METHODS

### 2.1 Apterostigma nest biology, collection, and laboratory maintenance

**FIGURE 1:**
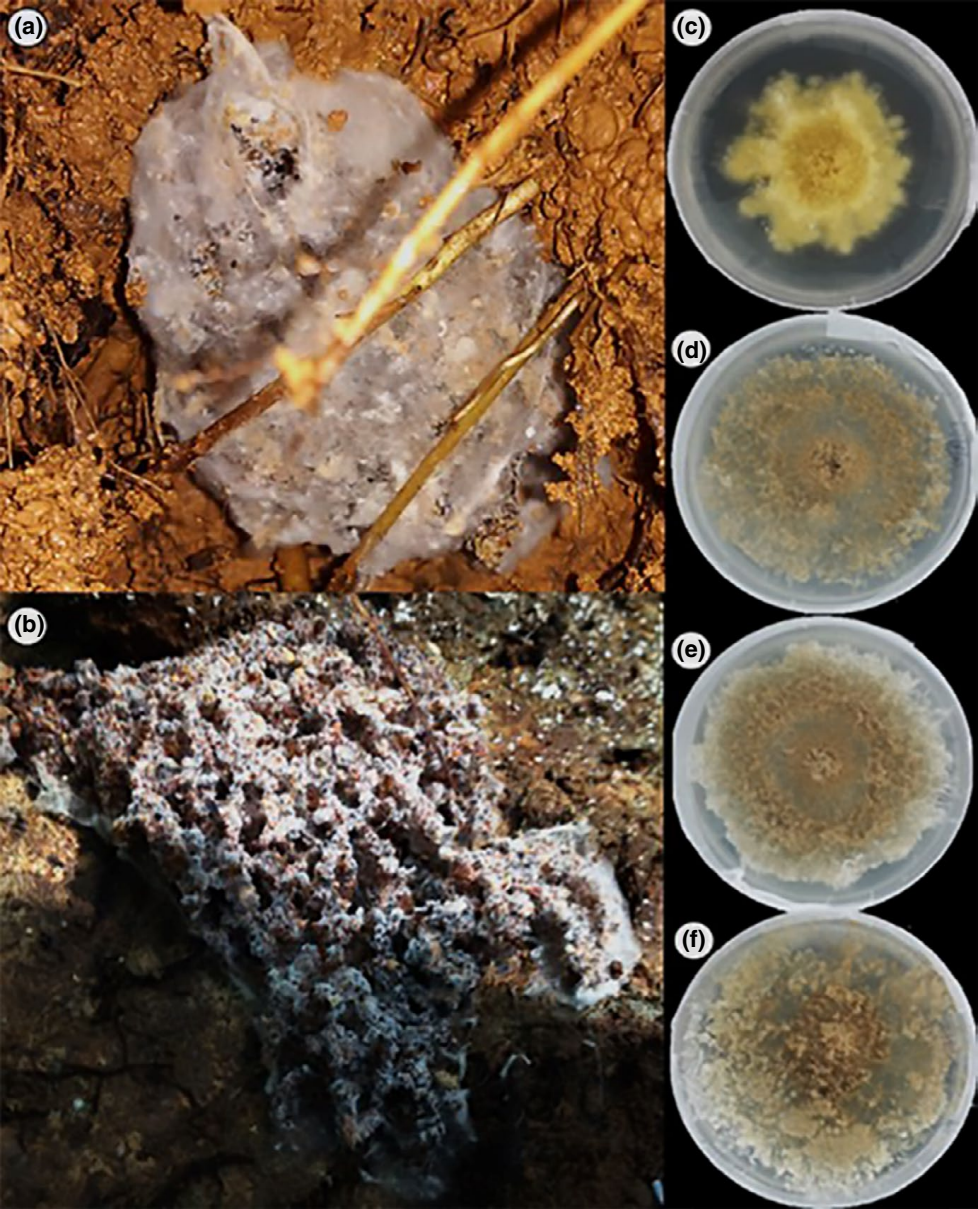
*Apterostigma* nests. (a) Nest of *Apterostigma dentigerum* with a mycelial veil; (b) nest of the *Apterostigma pilosum* without veil. *Escovopsis* morphotypes isolated from both *Apterostigma* species: (c) Y, yellow morphotype; (d) B1, brown morphotype; (e) B2, brown morphotype; and (f) B3, brown morphotype

*Apterostigma dentigerum* and *A*. *pilosum* are sympatric in central Panamá (Currie et al., 2003; Gerardo et al., 2006; González et al., 2019; Figure 1a,b), and both cultivate *M*. *velohortorum* (a.k.a., G2 fungi) (Pterulaceae: Basidiomycota) (Leal‐Dutra et al., 2020).

*Ap* *terostigma dentigerum* colonies are covered with a mycelial veil and hang under leaves, below leaf litter, or inside cavities of tree trunks, while *A*. *pilosum* colonies are recognized by the absence of a veil and are located inside decomposing logs, or under rotten wood on the ground. Both ant species cultivate their fungi on decomposing vegetation, insect detritus and frass (Fernández‐Marín et al., 2004; González et al., 2016).

*Apterostigma dentigerum* and *A. pilosum* colonies were collected in central Panamá, along Pipeline Road (9° 09′39.44″ N, 79 44′44.76″ W) and Plantation Road (9° 04′27.47″ N, 79 39′35.3″ W), in Soberania National Park. These colonies were located 20 to 50 cm apart, and none were suspected to be separate chambers of the same colony. Collected colonies were transferred to the laboratory and maintained at ambient room temperature in sterile Petri dishes (100 × 15 mm) containing sterile filter paper soaked in sterile water to retain humidity. Every two days, colonies were fed cornmeal and cleaned.

### 2.2 Diversity and prevalence of *Escovopsis* morphotypes in colonies of *Apterostigma* spp

To assess the prevalence and distribution of *Escovopsis* among host colonies of two ant species, we utilized two methods (M1 and M2) to maximize the likelihood of isolating parasite strains. First (M1), within five days of colony collection, from each of 32 *A*. *dentigerum* and 14 *A*. *pilosum* colonies, we isolated 50 pieces of fungal garden material (3–5 mm diameter) using sterilized forceps. Ten pieces were plated aseptically on a Petri dish containing potato dextrose agar (PDA, Scharlau culture media, 39 g/L) without antibiotics, and all five dishes per colony were incubated at room temperature for 10 days. Each day, the dishes were scanned for *Escovopsis* growth, which was transferred to a new PDA dish to obtain pure cultures. Second (M2), within five days of colony collection, 5 to 20 larger fungus garden pieces (~0.5 g) per colony (*N* = 12 for each species) without workers and brood were placed in sterile Petri dishes (100 × 15 mm), containing sterile, water‐soaked paper, sealed with parafilm. These garden fragments were incubated at room temperature and allowed to grow for 7 to 10 days. Each day, all dishes were scanned for *Escovopsis* growth, and fungi were transferred to a new PDA dish to obtain pure cultures.

### 2.3 Phenotypic characterization of prevalent *Escovopsis* morphotypes

*Characterization based on patterns of growth*. Four *Escovopsis* morphotypes isolated from *Apterostigma* species were classified based on spore color and growth rate. Hereafter, we refer to these morphotypes of *Escovopsis* based on color as Y (yellow conidia and fluffy mycelium, from *A*. *pilosum* colony); B1 (brown conidia, pulverulent mycelium with a single concentric ring of white conidia formed away from the inoculum, from *A. dentigerum* colony); B2 (brown conidia, pulverulent mycelium with a single concentric ring of white to light brown conidia formed away from the inoculum, from *A*. *dentigerum* colony); and B3 (dark brown conidia, pulverulent mycelium with a single concentric ring of light to dark brown conidia formed away from the inoculum, from *A*. *pilosum* colony) (Figure 1c‐f). As an outgroup, given the specificity reported for the *Escovopsis*–fungal cultivar symbiosis (Birnbaum & Gerardo, 2016; Gerardo et al., 2006; Taerum et al., 2007), we isolated a fifth morphotype of *Escovopsis* from *Trachymyrmex zeteki* (TB) (cottony hard mycelium growth and brown colored conidia) (Appendices S1–S2).

To obtain micrographs of *Escovopsis* conidia using scanning electron microscopy, we selected a single strain randomly from each of the five morphotypes. We grew the strains on PDA for seven days at room temperature and fixed their fungal structures with osmium tetroxide vapor. To determine the fungal growth rates of *Escovopsis* morphotypes, we collected individual fungal pieces of *Escovopsis* conidia from pure culture using a sterilized inoculating needle with a looped end (1 mm^2^). We placed each piece in the center of a Petri dish (100 × 15 mm) containing PDA without antibiotics. We plated ten pieces per morphotype. The dishes were sealed with parafilm and incubated at 12:12 light:dark and at room temperature (~27°C) for 20 days. Each Petri dish then was photographed using a Nikon Coolpix camera. Total area of growth was analyzed using ImageJ v1.4 (Schneider et al., 2012).

*Characterization based on chemical profiling*. To determine the chemical profiles of the five *Escovopsis* morphotypes, we grew 200 PDA dishes/morphotype at room temperature for 20 days. All the fungal growth on agar was cut into small pieces with a sterile spatula and transferred to Erlenmeyer flasks (500 ml). We added 500 ml of EtOAC (ethyl acetate, Merck) to each flask and wrapped the flasks with aluminum foil. The fungal agar pieces were extracted by vaporization (Rotavapor R‐215) and ultrasonic bath (Branson 1510, Sonicator). The supernatant, containing the organic compounds, was transferred to a new vial, lyophilized, and redissolved in 500 μl of methanol assisted by ultrasonication for 20 min. The aliquots were filtered into HPLC vials using a 0.45‐μm polytetrafluoroethene (PTFE) filter. The molecular weight of metabolites of five *Escovopsis* morphotypes was analyzed by UHPLC‐ESI‐Q‐TOF‐MS/MS. Analyses were performed using ultra‐high‐performance liquid chromatography (UHPLC) with a diode array detector and maXis 3G QTOF mass spectrometer (MS) (Bruker Daltonics, Bremen, Germany) equipped with an electrospray source (ESI) and connected to an Ultimate 3000 UHPLC system (Dionex, Sunnyvale, USA) equipped with a Kinetex 2.6‐μm C18, 100 mm × 2.1 mm column (Phenomenex, Torrance, CA) to accurately measure the molecular weight of the metabolites. MS was performed in ESI+, at the scan range m/z 100–1000, with a mass accuracy < 1.5 ppm. UV/VIS spectra were collected at wavelengths from 200 to 700 nm. For each strain, presence or absence of 171 chemical compounds, all with low molecular weight, was coded into a PAUP data matrix using binary characters. This binary matrix was used to construct a phylogeny based on chemical similarities among the strains. CIPRES Science Gateway (Miller et al., 2010) was used to conduct a maximum‐likelihood (ML) phylogenetic analysis using RAxML 8.2.9 (Stamatakis, 2014) under the BINGAMMA model. Bootstrap analysis was performed with 1000 Ml bootstrap replicates on the data matrix. Data files for these chemical analyses are deposited in Dryad (accession number below).

### 2.4 Placement of *Apterostigma*‐associated *Escovopsis* within the broader *Escovopsis* phylogeny

DNA from nine *Escovopsis* strains (four *Escovopsis* morphotypes from each of the two *Apterostigma* ant species and one *Escovopsis* strain from *T. zeteki*) was extracted from mycelia of fresh cultures on PDA following established protocols (Augustin et al., 2013). PCRs were performed independently for each extracted DNA sample for the internal transcribed spacer (ITS) region.

#### 2.4.1 PCR and Sequencing analysis

PCRs were performed independently for each extracted DNA sample to amplify the internal transcribed spacer (ITS) region using primers ITS5 (5′GGAAGTAAAAGTCGTAACAAGG3′) and ITS4 (5′TCCTCCGCTTATTGATATGAC3′; Augustin et al., 2013). All PCRs were performed in a total volume of 24.5 µl containing 20 to 100 ng of genomic, high‐quality DNA; buffer 10× (50 mM KCl, 10 mM Tris‐HCl); 1.5 mM MgCl2; 0.2 µM of each dNTP; 1 U of Taq DNA polymerase; and 10 µM of each primer. All reactions were conducted in a Labinco thermocycler.

PCR and sequencing conditions consisted of initial denaturation at 95°C for 5 min; 30 cycles of 95°C for 45 s, 50°C for 45 s, and 72°C for 1.5 min; and a final extension at 72°C for 10 min. PCR products were cleaned with the QIAquick PCR Purification Kit (Qiagen, Hamburg, Germany) following the supplier's instructions and sequenced by Eurofins Genomics (Germany). Contigs were assembled in the software Geneious Prime 2019.0.3 (https://www.geneious.com; Kearse et al., 2012) and queried using NCBI tblastn (Altschul et al., 1990) to find the closest known relatives. The sequences are deposited in GenBank under accessions MK936049–MK936057. For the phylogenetic analysis, we included our nine *Escovopsis* strains and representative strains of ten *Escovopsis* species described in previous studies (Augustin et al., 2013; Masiulionis et al., 2015; Meirelles et al., 2015; Montoya et al., 2019). *Hypomyces* spp. and *Trichoderma harzianum* were used as outgroups. The sequences were aligned with Multiple Alignment using the Fast Fourier Transform (MAFFT v7.017) (Katoh & Standley, 2013). The phylogeny was reconstructed by two different methods: maximum‐likelihood phylogenies were constructed using IQtree (Nguyen et al., 2015), with models of molecular evolution selected on the basis of Bayesian information criterion (BIC) scores calculated with jModelTest (Darriba et al., 2012). Support for individual branches was assessed using 1000 ultrafast bootstraps (Hoang et al., 2018) and Bayesian inference (BI) using MrBayes v. 3.2.2. (Ronquist et al., 2012).

### 2.5 In vitro *Escovopsis*–cultivar interactions

We conducted confrontation bioassays between the *Escovopsis* strains and cultivars of *A. dentigerum* (CAd.) and *A. pilosum* (CAp.) to assess whether the parasites vary in their ability to grow in the presence of alternative cultivars. We first plated small pieces of cultivar mycelium on the edges of PDA Petri dishes (100 × 15 mm) and allowed them to grow for 20 days. Then, we used a sterilized cork borer to cut 0.5 cm diameter plugs of fresh mycelium of *Escovopsis* on PDA. We placed plug on the opposite side of a Petri dish from the cultivar. We carried out four replicates of each combination: CAd.‐Y and CAp.‐Y; CAd.‐B1 and CAp.‐B1; CAd.‐B2 and CAp.‐B2; CAd.‐B3 and CAp.‐B3; and CAd.‐TB and CAp.‐TB; and three replicates of controls with each *Escovopsis* strain alone (Y Ct, B1 Ct, B2 Ct, B3 Ct, and TB Ct). All plates were monitored and photographed every five days for 20 days, and the growth area of *Escovopsis* (in cm^2^) was measured using ImageJ 1.4v (Schneider et al., 2012; Appendix S3). We performed a two‐way ANOVA with interaction effect between treatment (fungal cultivar isolated from each ant species) and *Escovopsis* strains (five morphotypes). All analysis and plots were performed with the statistical software R (R Development Core, 2008). Two‐way ANOVA was performed using the aov() function; normality and homoscedasticity of the data were analyzed using the shapiroTest() and leveneTest() functions. Pairwise comparisons were adjusted using the Tukey HSD function.

### 2.6 Behavior of ants in response to *Escovopsis* infections of their gardens

To evaluate antiparasitic behavioral defenses (e.g., fungal grooming; Currie & Stuart, 2001) in response to alternative *Escovopsis*, we scored behavior of ants from 22 ant colonies not previously inoculated with *Escovopsis* (10 colonies of *A*. *dentigerum* and 12 colonies of *A*. *pilosum*). We conducted experiments using entire colonies. Prior to infection, we cultured a representative of each of the five *Escovopsis* morphotypes on PDA media at room temperature for 20 days. We then weighed each colony and recorded the number of ants. We cut 0.5 cm PDA plugs of *Escovopsis* conidia as described above. The *Escovopsis* conidia were inoculated randomly in five areas of the fungus garden (five morphotypes per dish), maintaining a distance of ∼1 cm between pieces. The *Escovopsis* plugs were placed gently on the fungus garden, applying conidia to the garden. We inoculated each of the fungus gardens with 4.4 ± 0.17 × 10^6^ dry conidia (mean ± *SD*) of each of the morphotypes of *Escovopsis* and waited five minutes for acclimation of the workers. Using stereomicroscopy (Leica 10x, with a dim, not hot light), we recorded the number of workers fungal grooming the areas with each *Escovopsis* morphotype every 10 min for two hours; grooming behaviors involved scanning the fungal garden surface to remove contaminants (Currie & Stuart, 2001; Fernández‐Marín et al., 2013). The Petri dishes were gently moved, without affecting the behavior of the ants. For each nest, we summed the total number of grooming events observed in each of the five infected garden regions. We assessed whether the ant species differed in their propensity to groom infections of each *Escovopsis* type using multinomial logistic regression via the multicomp() function in the nnet package of R. Significance was assessed using a likelihood ratio test.

To better understand the outcome of grooming in response to *Escovopsis* garden infection, we evaluated the accumulation of microbes in infrabuccal pellets after *Escovopsis* infection of their gardens. Infrabuccal pellet comprised of detritus and other materials that are packed into a specialized pocket on the head after ant grooming behavior. They are then discarded to trash piles (Fernández‐Marín et al., 2006; Little et al., 2006). We formed five subcolonies, each consisting of 0.3 g of fungus garden and 10 workers, from each of five colonies of *A*. *dentigerum* and 10 colonies from *A*. *pilosum*, and placed them onto separate, sterile Petri dishes (100 × 15 mm). We cut 0.65 cm PDA plugs covered with *Escovopsis* conidia as before. The fungus garden from each subcolony was inoculated with 5.3 ± 0.47 × 10^6^ conidia (mean ± *SD*) by rubbing a sterilized swab of *Escovopsis* conidia onto the center of the garden, one morphotype per subcolony. Eight hours after inoculation, we removed the workers, and all infrabuccal pellets were collected and plated on PDA. The infrabuccal pellets were incubated at room temperature to score germination of *Escovopsis*, antibiotic‐producing actinomycete bacteria, mutualistic cultivar, and other nonmutualistic fungi. We performed a one‐way MANOVA to evaluate the effect of host ant species, *Escovopsis* morphotype, and their interaction. Data were transformed (log + 1) when the assumption of normality was violated, and homoscedasticity of the data was checked with the leveneTest() function. Outliers were detected using box plots and removed from the analysis. To detect the global effect of each variable, we conducted a univariate one‐way ANOVA using the aov() function.

## 3 RESULTS

### 3.1 Diversity and prevalence of *Escovopsis* morphotypes in sympatric *Apterostigma* spp

**FIGURE 2:**
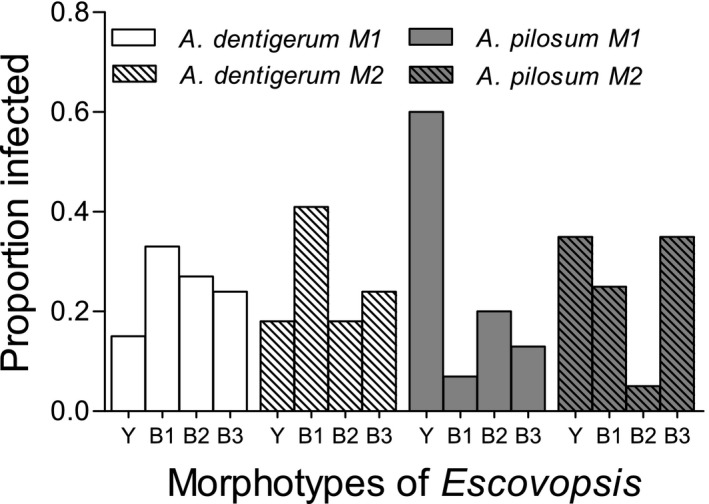
Proportion of colonies infected with four morphotypes of *Escovopsis* isolated from *A*. *dentigerum* and *A*. *pilosum* colonies using small portions of fungal cultivar mass (methodology M1, solid bars) or using whole fungal cultivar mass (methodology M2, striped bars)

We isolated 48 *Escovopsis* isolates from a total of 2,300 small pieces of fungal garden (M1) (prevalence = 2.1%), including 33 from *A*. *dentigerum* and 15 from *A*. *pilosum* colonies. We isolated 37 *Escovopsis* isolates from a total of 219 larger pieces of fungal garden (M2) (prevalence = 16.8%), including 17 from *A. dentigerum* and 20 from *A. pilosum* colonies. Through both methods, we identified a total of four *Escovopsis* morphotypes, which we classified based on spore color and general growth form as Y (yellow), B1 (brown 1), B2 (brown 2), and B3 (brown 3). The two methodologies (M1: isolation from small garden pieces; M2: isolation from large gardens pieces) differed significantly in their abilities to isolate these morphotypes (*N* = 85, *X* ^2^ = 3.37 *df* = 3, *p* =.34). The frequency of *Escovopsis* morphotypes isolated differed significantly between ant species (*N* = 85, *X* ^2^ = 10.8, *df* = 3, *p* =.013); the two methods gave overlapping results for both species, though less so for *A. pilosum*. Brown morphotypes (B1, B2, and B3) were more commonly isolated from fungus gardens of *A. dentigerum*, while the yellow morphotype (Y) was more frequently isolated from fungus gardens of *A. pilosum* (Figure 2). Coinfections were seen in 5 of the 40 *A. dentigerum* and 6 of 26 *A. pilosum* colonies that were infected with *Escovopsis*.

### 3.2 Phenotypic characterization of prevalent *Escovopsis* morphotypes

#### 3.2.1 Characterization based on growth form

**FIGURE 3:**
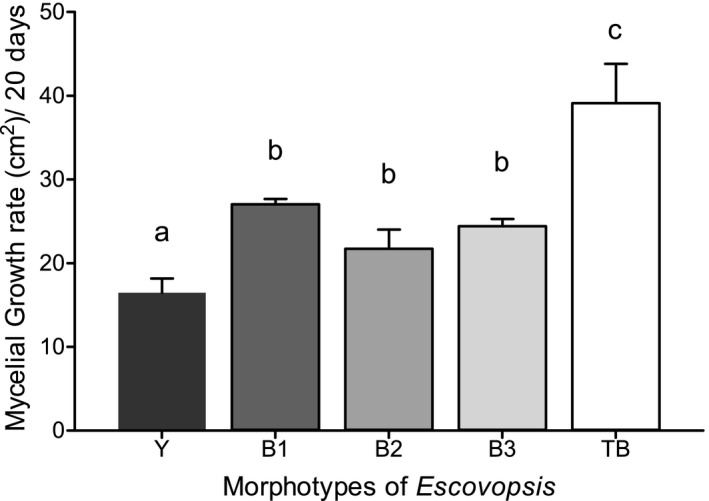
Area of growth exhibited by five morphotypes of *Escovopsis* fungal parasite cultivated on PDA in the absence of cultivars after 20 days. Different letters above columns indicate significant differences between morphotypes based on pairwise comparisons (*post hoc* Tukey's tests; *p* <.05). *Escovopsis* morphotypes isolated from *A*. *dentigerum* (B1, B2), *A*. *pilosum* (Y, B3).d and *Trachymyrmex zeteki* (TB)

All four morphotypes of *Escovopsis* grew well on PDA, but they showed significant differences in growth after 20 days (*F*_4,10_ = 37.46, *p* <.0001). In vitro, brown *Escovopsis* morphotypes from *Apterostigma* (B1, B2, B3) grew significantly more than the Y morphotype *Escovopsis* in the same time period but did not differ in growth rate from one another; the TB morphotype grew significantly more than all four of the morphotypes from *Apterostigma* colonies (Figure 3).

#### 3.2.2 Characterization based on chemical profiling

**FIGURE 4:**
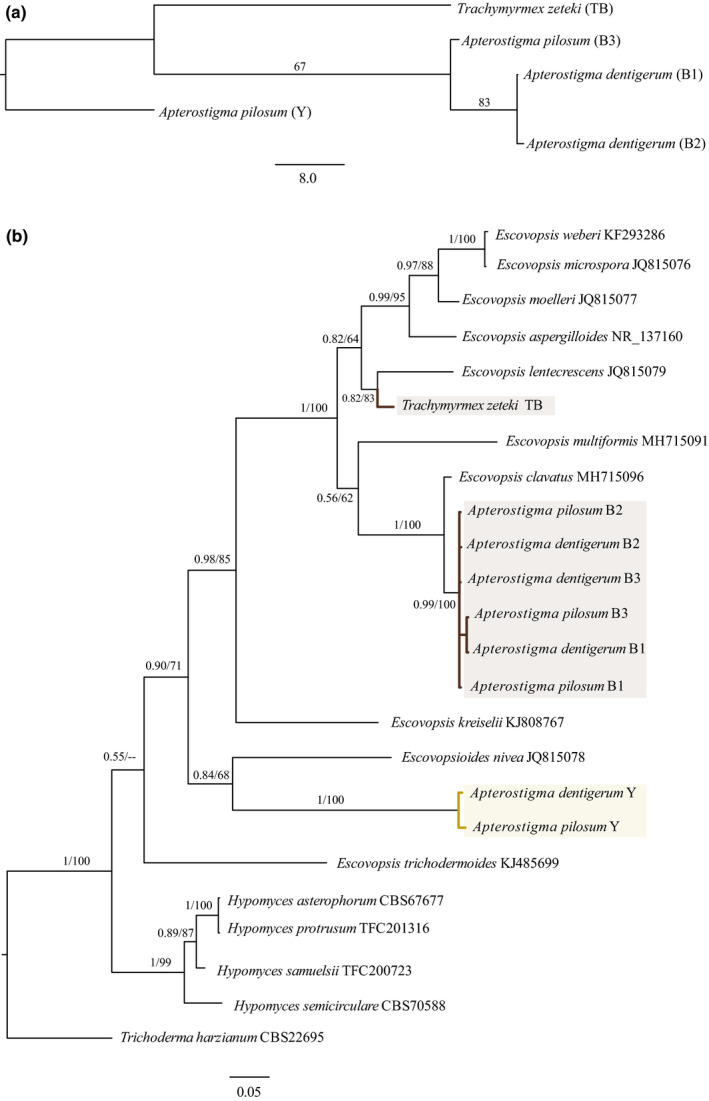
Phylogenetic trees of *Escovopsis* fungal parasites based on chemical and molecular similarities. (a) Phylogeny of five morphotypes based on a binary alignment of chemical profiles (171 chemical ions). Tree was reconstructed under maximum‐likelihood inference, and bootstrap values are depicted at the nodes. Scale bar indicates substitutions per site. (b) Bayesian phylogeny of nine *Escovopsis* isolates of the five morphotypes from *Apterostigma dentigerum*, *Apterostigma pilosum,* and *Trachymyrmex zeteki* together with other relevant *Escovopsis* spp. based on internal transcribed spacer gene sequences. Each parasite strain is either indicated by the species name of the ant host garden from which *Escovopsis* was isolated (our data) or, in cases where the parasite has been named to the named to the species level by genus and species. The trees reconstructed by BI and ML analyses were similar in topology. The numbers on branches indicate the posterior probabilities and the ultrafast bootstrap support values, respectively. *Hypomyces* spp. and *Trichoderma harzianum* were used as outgroups. Only bootstrap values higher than 50% are shown. For sequences obtained from GenBank, accessions are listed after each taxon name. Scale bar indicates 0.05 substitutions per site

We detected 171 ion signals across chemical profiles based on their molecular weights. Of these 171 signals, we identified 16 based on mass as belonging to a group of cyclic peptides, suggesting the presence of a series of diketopiperazine‐type compounds in the extracts produced by these *Escovopsis* (Appendix S4). Phylogenetic analyses based on chemical profiles yielded a single most parsimonious tree (Figure 4a). Bootstrap analyses strongly supported monophyly among *Escovopsis* brown morphotypes, while the yellow *Escovopsis* morphotype formed a distinct clade.

### 3.3 Placement of *Apterostigma*‐associated *Escovopsis* within a broader *Escovopsis* phylogeny

Based on ITS sequences, the brown *Escovopsis* morphotypes isolated from *A*. *dentigerum* and *A*. *pilosum* colonies (B1, B2, B3) formed a single clade, distinct from the brown TB morphotype from *T*. *zeteki*. The two isolates of the Y morphotype also clustered together. The brown morphotypes were most genetically similar to *E. clavatus*, while the Y morphotype was most similar to *Escovopsoides nivea*, though support for this placement was low, and thus, we still use *Escovopsis* in reference to all isolates. The trees reconstructed by BI and ML analyses were similar in topology, BI posterior probabilities, and ML ultrafast bootstrap support values are shown in Figure 3b.

### 3.4 In vitro *Escovopsis*–cultivar interactions

**FIGURE 5:**
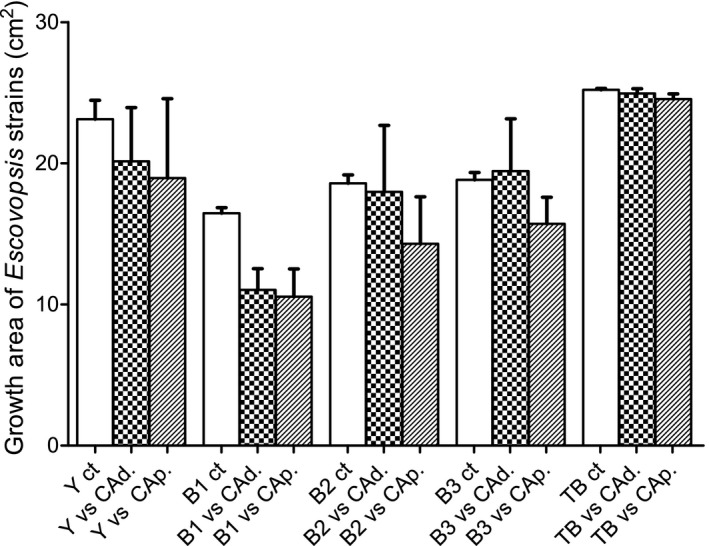
In vitro growth of *Escovopsis* morphotypes in the presence and absence of host fungal cultivars. Letters Y, B1, B2, B3, and TB represent five *Escovopsis* morphotypes grown in the presence of fungal cultivars of *Apterostigma dentigerum* (CAd.) or *Apterostigma pilosum* (CAp.), or a blank control (Ct). Mean + *SEM*

*Escovopsis* morphotypes significantly varied in area of growth during in vitro bioassays (Figure 5, Table 1, Appendices S5–S6), with the TB isolate growing the most and the B1 isolate the least. The cultivar treatment (i.e., *A. dentigerum* cultivar, *A. pilosum* cultivar, or no cultivar) also had a significant impact on *Escovopsis* growth, with signs of the cultivars suppressing growth of all *Escovopsis* morphotypes except TB. There was no significant interaction between *Escovopsis* morphotype and cultivar treatment, and in pairwise comparisons, there were no significant differences in *Escovopsis* mycelial growth based on the origin of the cultivar (i.e., from *A. dentigerum* or *A. pilosum* colonies).

**TABLE 1:** Two‐way ANOVA assessing the effect of fungal cultivar type and *Escovopsis* morphotype on in vitro growth of *Escovopsis*

Source	Partial sum of squares	Degrees of freedom	Mean squares	*F* statistic	*p* Value
Fungal cultivar (FC)	114.1	2	57.06	7.28	.002**
*Escovopsis* morphotype (EM)	945.0	4	236.26	30.14	1.36^e−11^***
FC * EM	59.3	8	7.41	0.95	.49
Residuals	313.5	40	7.84		

**p <.01 and ***p <.001.

### 3.5 Behavior of ants in responses to *Escovopsis* infections of their gardens

**FIGURE 6:**
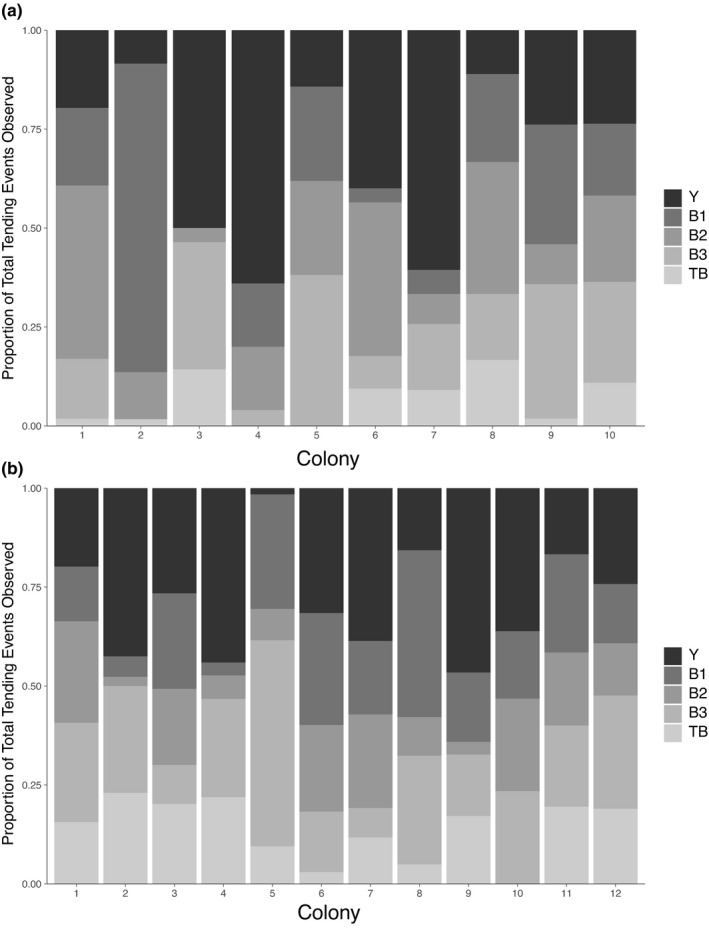
Proportion of *A*. *dentigerum* (a) and *A*. *pilosum* (b) worker ants engaging in fungal grooming behavior in garden regions infected with different *Escovopsis* strains during coinfections with five *Escovopsis* morphotypes. Each bar represents one colony

In response to inoculation of colonies with the conidia of multiple *Escovopsis* strains, the ant species exhibited significant differences in the proportions of ants grooming the areas of garden infected by different parasites (likelihood ratio test: *X* ^2^ = 67.04, *p* = 7 × 10^–14^). Across all 22 colonies (Figure 6), an average of 33%, 20%, 18%, 21%, and 10% of grooming events observed in a colony were of the Y‐, B1‐, B2‐, B3‐, and TB‐infected areas, respectively. The most striking difference for both ant species was in their reduced propensity to groom the TB *Escovopsis* (*A. dentigerum* colonies, 7% of grooming events per colony involved the TB‐infected area, and 13% for *A. pilosum*).

**FIGURE 7:**
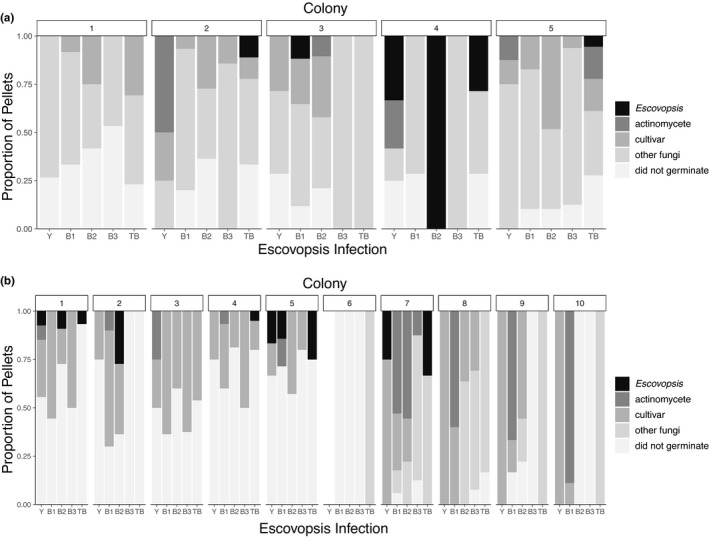
Emergence of microbes from infrabuccal pellets collected from (a) *A. dentigerum* colonies and (b) *A. pilosum* colonies. Colonies were subdivided into five subcolonies, each receiving a different *Escovopsis* infection (Y, B1, B2, B3, or TB, as in previous figures). Emergence of microbes was recorded from recovered pellets (*n* = 0 to 29 pellets per subcolony, mean = 12, *SD* = 7); no pellets were recorded from colony 6 when infected with Y morphotype *Escovopsis*. Colonies vary significantly in the types of microbes that emerge from pellets, and the *Escovopsis* morphotype with which the subcolony was infected also influences microbial emergence from pellets

After eight hours of infection of subcolonies using inoculations of single morphotypes of *Escovopsis*, we collected and plated 271 infrabuccal pellets produced by workers in five colonies of *A*. *dentigerum* and 227 pellets produced by workers in 10 colonies of *A*. *pilosum*. The frequencies with which *Escovopsis*, actinomycete bacteria, the fungal cultivar, and other fungi grew from the infrabuccal pellets differed significantly between host ant species (Pillai's Trace = 0.689, *F*_1,65_ = 34.5, *p* <.0001), between *Escovopsis* strains with which the subcolony was infected (Pillai's Trace = 0.563, *F*_4,65_ = 2.66, *p* <.001), and based on the interaction between them (Pillai's Trace = 0.419, *F*_4,65_ = 1.90, *p* =.02; Table 2, Figure 7).

**TABLE 2:** Summary of the multivariate analysis of variance (MANOVA) showing the effects of host ant species and *Escovospsis* infrabuccal pellet‐derived microbes

Source	Pillai´s trace	Degrees of freedom	*F* statistic	*P* value
Host ant species (HAS)	0.69	1	34.50	3.82^–15***^
*Escovopsis* morphotype (EM)	0.56	4	2.6	.0006^***^
HAS * EM	0.42	4	1.9	.02

**p <.01 and ***p <.001.

## 4 DISCUSSION

### 4.1 Diversity and frequency of Escovopsis morphotypes between ant congeners

Our results highlight that the fungal cultivars of colonies of two closely related, sympatric *Apterostigma* ant species are infected by the same *Escovopsis* morphotypes. Sympatric colonies of *Cyphomyrmex* spp. have also been shown to be infected by similar *Escovopsis* morphotypes, with those species that grow more genetically similar cultivars being infected with more genetically similar parasites (Birnbaum & Gerardo, 2016; Gerardo et al., 2004). Similarly, sympatric colonies of *Atta* spp. and *Acromyrmex* spp. leaf‐cutting ants are attacked by the same *Escovopsis* parasites (Meirelles, Solomon, et al., 2015; Taerum et al., 2007).

There are, however, differences in the prevalence of *Escovopsis* strains infecting colonies of *Apterostigma dentigerum* and *A. pilosum*, in the degree to which cultivars suppress growth of the pathogen species, and in the degree to which pathogen species stimulate ant defensive behaviors.

Colonies of both *A*. *dentigerum* and *A*. *pilosum* were frequently infected with multiple *Escovopsis* strains (11.4% and 23.07%, respectively), a phenomenon also observed in relation to colonies of other fungus‐growing ant species (Taerum et al., 2010). Based on theory, coinfecting parasites are expected to compete with each other for host resources (competitive suppression) but incidentally may together overwhelm host defenses (Abdullah et al., 2017; Bashey, 2015). The impact of coinfection can be influenced by the degree of genetic similarity between coinfecting parasites (Read & Taylor, 2001) and by changes in parasite behavior in the presence of coinfecting parasites (Rayner, 1991). If *Escovopsis* coinfections are characterized by competitive suppression, then this could lower infection virulence overall, which is consistent with observations that seemingly healthy colonies can harbor low‐level chronic infections for years (N. Gerardo, per obs.). Alternatively, if different parasites require alternative forms of defense, coinfection may generally weaken defenses and then multiple infections could overwhelm a colony.

### 4.2 The utilization of multifaceted, specific defense strategies

In response to *Escovopsis* infection, fungus‐growing ants groom their gardens and apply antimicrobial compounds. This behavior facilitates removal of infectious parasite spores. The congeneric *Apterostigma* ants groomed garden areas infected by some parasite species more than others. Both species tended to groom areas infected with the yellow *Escovopsis* morphotype more than areas infected with the brown *Escovopsis* morphotype, but this was significant only for *A. pilosum*. Most strikingly, the ants tended the garden area infected with TB *Escovopsis*, which they may not encounter in nature much less the garden areas with other *Escovopsis*. The ants’ differential responses to different parasites suggest that the ants can discriminate between different *Escovopsis* strains as previously suggested (Goes et al., 2020). However, we do not know whether ants' attention to particular morphotypes can be modified by adding or removing other *Escovopsis* morphotypes or pathogens. Previous in vivo infection experiments studies demonstrated that leaf cutter ants in the genera *Atta* and *Acromyrmex* adjust their hygienic defensive strategies (e.g., metapleural glands grooming) to respond to distinct fungal conidia (Fernández‐Marín et al., 2013), and adjust the quantity of their antifungal metapleural gland secretions in response to different pathogens (Fernández‐Marín et al., 2015; Yek et al., 2012). These findings, coupled with our own, indicate that fungus‐growing ants are able to differentially respond to conidia of distinct fungal types, including *Escovopsis* spp. that threaten their gardens’ health.

This specificity of the defensive response requires a mechanism for the ants to recognize and distinguish different *Escovopsis* strains (Currie & Stuart, 2001; Fernández‐Marín et al., 2009), but nothing is known of the sensory mechanisms for detection and discrimination. We hypothesize that this recognition could be driven by differential parasite chemistry, as the ants exhibited similar frequencies of fungal grooming toward *Escovopsis* that were chemically similar (Figure 4a,b). However, aspects of parasite morphology, growth, and other phenotypes could also modulate the behavioral responses of the ant hosts.

In a separate experiment, we isolated microbes from infrabuccal pellets to assess the degree to which the ants may be utilizing two defensive strategies, grooming (Currie & Stuart, 2001) and actinomycete‐based antibiotic inhibition (Currie, Scott, et al., 1999). Infrabuccal pellets reflect material that worker ants have removed from the garden or microbes being used to actively kill pathogens (Fernández‐Marín et al., 2006). Presence of *Escovopsis* in the pellets likely reflects active grooming of infected areas. In terms of antibiotic suppression, previous research has shown that upon infection with *Escovopsis*, infrabuccal pellets are more likely to contain actinomycete bacteria that produce antibiotics that can suppress *Escovopsis* growth (Little et al., 2006). Thus, increased proportions of pellets containing these bacteria in response to some *Escovopsis* and not others could suggest that some *Escovopsis* stimulate a stronger actinomycete‐based antimicrobial response. The disease management implications of the presence of cultivar and nonmutualistic fungi in the pellets is less clear. Interestingly, *Escovopsis* morphotype, ant species, and the interaction between the two all had significant impacts on the microbial composition of isolated pellets, suggesting that the ants use defenses differently based on the type of parasite.

In addition to the ant‐ and bacteria‐derived defenses, fungal cultivars produce compounds with antifungal properties that can inhibit *Escovopsis* fungal growth or spore germination (Oh et al., 2009; Wang et al., 1999). The result of our fungal cultivar versus *Escovopsis* interaction experiments showed no effect of the fungal cultivars on growth of the *Escovopsis* TB morphotype, but inhibition of both the yellow and brown *Escovopsis* morphotypes from *Apterostigma* spp. The ants also showed reduced behavioral response to the TB morphotype compared with the yellow and brown *Escovopsis* strains. This is consistent with the hypothesis that both the ants and their mutualistic fungi are under selection to maintain defenses that suppress the parasites that infect them in nature, and it is also consistent with previous work indicating that both ant genotype and cultivar lineage influence the outcome of *Escovopsis* infections (Kellner et al., 2018). However, previous work suggested that cultivars are more likely to inhibit the growth of non‐native pathogens than the pathogens that attack them in nature (Birnbaum & Gerardo, 2016; Custodio & Rodrigues, 2018). Overall, patterns of these bioassays may be highly dependent on the parasite and host strains utilized, which would be consistent with a model of ongoing coevolution. Thus, difference across studies, particularly when few host and parasite combinations are utilized (as here), is not surprising.

## 5 CONCLUSIONS

*Apterostigma* ants utilize multiple defenses against *Escovopsis* infections, but the prevalence of *Escovopsis* infections suggests that the parasites possess mechanisms to overcome these defensive strategies, establish infections, and prevent clearance at least some colonies. The utilization of multiple defensive strategies, and the differential utilization of different strategies between congeneric ant species in sympatry, might help reduce the likelihood of the evolution of resistance of *Escovopsis* against colony‐level host defenses. Studies integrating different approaches at distinct scales are needed to better understand how variation in colony defenses within populations of more than one host species shape ecology and evolution dynamics of these infections. To further elucidate population‐level disease dynamics and defense, more detailed taxonomic and molecular investigations of *Escovopsis* are needed (see Augustin et al., 2013; Masiulionis et al., 2015; Meirelles, Montoya, et al., 2015; Montoya et al., 2019). In particular, future research should study geographic patterns of host–parasite association in this system and coupled this work with experimental studies of coinfection, infection outcomes, and defense utilization.

## CONFLICT OF INTEREST

None declared.

## AUTHOR CONTRIBUTION

**Yuliana Christopher Herrera:** Conceptualization (equal); Data curation (equal); Formal analysis (equal); Funding acquisition (equal); Investigation (equal); Methodology (equal); Project administration (equal); Writing‐original draft (equal); Writing‐review & editing (equal). **William Wcislo:** Funding acquisition (equal); Writing‐review & editing (equal). **Sergio Martinez‐Luis:** Formal analysis (equal); Methodology (equal); Writing‐original draft (equal); Writing‐review & editing (equal). **William O H Hughes:** Funding acquisition (equal); Writing‐review & editing (equal). **Nicole Gerardo:** Formal analysis (equal); Investigation (equal); Writing‐review & editing (equal). **Hermogenes Fernández‐Marín:** Conceptualization (equal); Formal analysis (equal); Funding acquisition (equal); Investigation (equal); Methodology (equal); Writing‐original draft (equal); Writing‐review & editing (equal).

## Supporting information

Supplementary Material

## Data Availability

DNA sequences are available in GenBank accessions MK936049–MK936057. Data supporting the results presented here are available at Dryad Digital Repository (https://doi.org/10.5061/dryad.gb5mkkwmx).
