# Supplementary material for: Disease management in two sympatric Apterostigma fungus‐growing ants for controlling the parasitic fungus Escovopsis
Source: Ecol Evol. 2021 May 2;11(11):6041–52. doi: 10.1002/ece3.7379 (PMC8207340; doi:10.1002/ece3.7379)
Supplement: Supplementary file 1 — Supplementary Material [file ECE3-11-6041-s001.docx]

**APPENDICES**


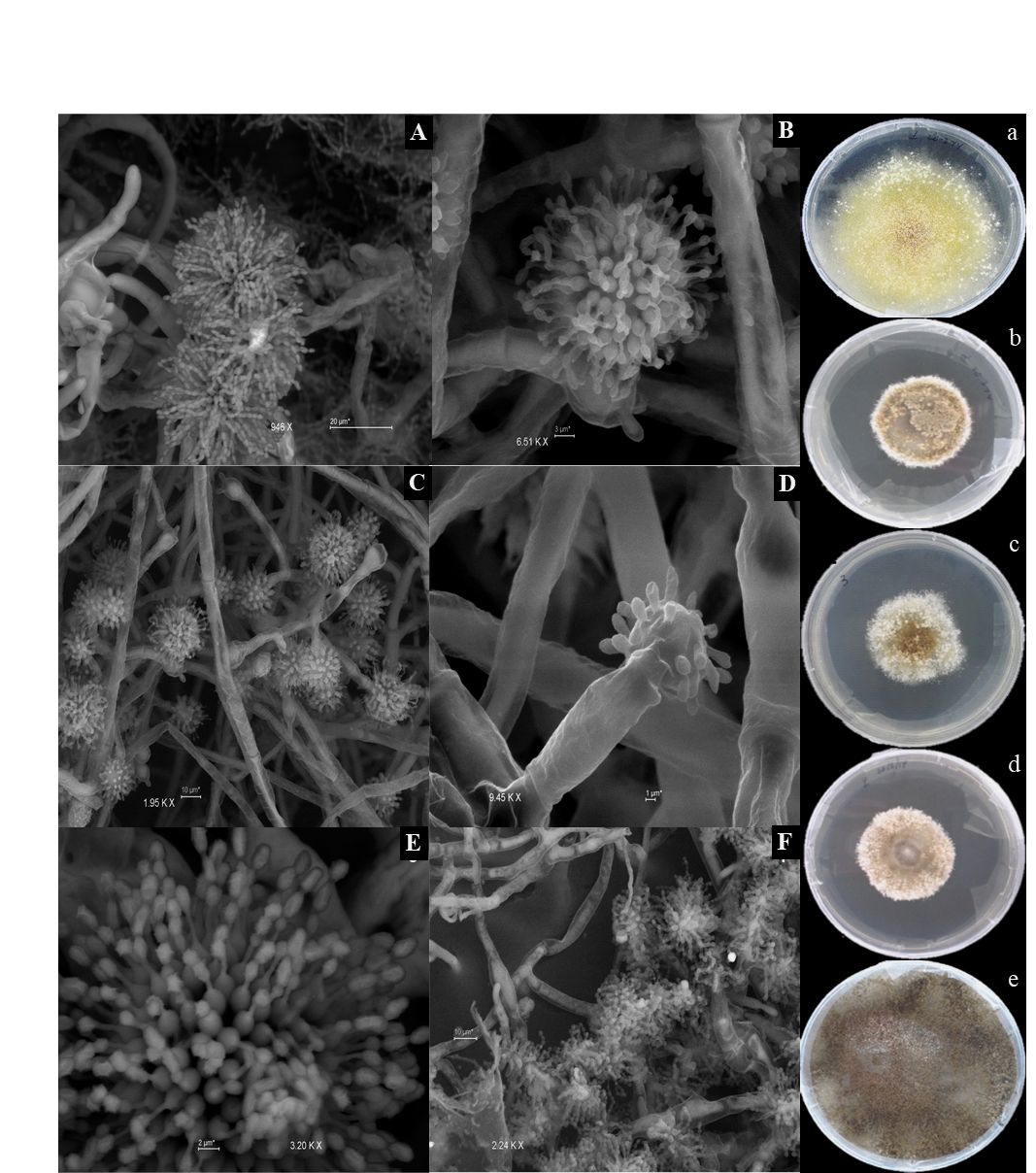
**Appendix S1** Scanning electron microscopy images of five *Escovopsis* morphotypes isolated from *A. dentigerum* (B1, B2), *A. pilosum* (Y, B3) and *T. zeteki* (TB). Isolates were grown on PDA at 25°C for seven days*. (A)* *Escovopsis* Y, showing detail of conidial morphology, ornamentation and long chains of conidia (scale bar = 20 µm), and its (a) growth form; *(B)* *Escovopsis* B1 and *(C) Escovopsis* B2 conidiophores’ growth patterns on aerial mycelia (scale bars = 5, 10 µm, respectively) and their (b, c) growth form, respectively; (*D*) *Escovopsis* B3, showing branching and globose vesicle formation (scale bar = 1 µm) and its (d) growth form; (*E*) *Escovopsis* TB, showing details of conidiogenesis and spore morphology (scale bar = 10 µm) and its (e) growth form. *(F)* *Escovopsis* Y, showing phialides produced (scale bar = 2 µm).

**Appendix S2** Growth area measurement recorded from *Escovopsis* morphotypes, after 20 days on PDA at room temperature. Petri dishes showing, from left to right, measurement of *Escovopsis* Y (Yellow), *Escovopsis* B1 (Brown 1), *Escovopsis* B2 (Brown 2), *Escovopsis* B3 (Brown 3) and *Escovopsis* TB (isolated from *Trachymyrmex zeteki*).


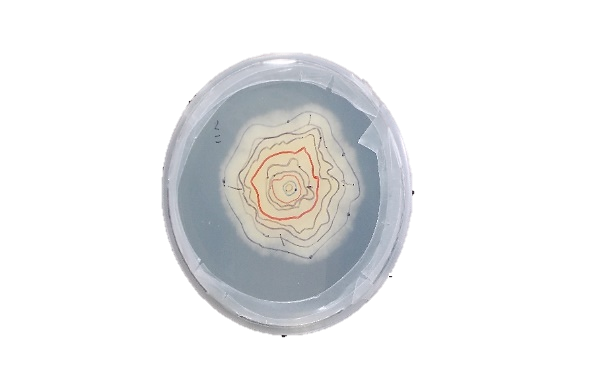

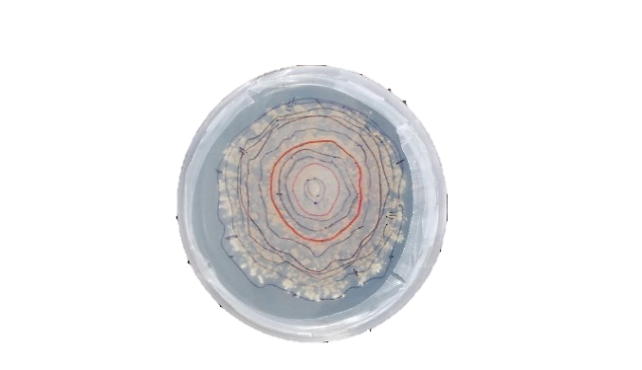

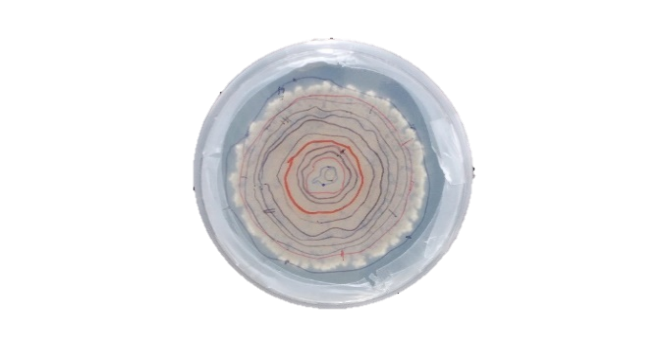

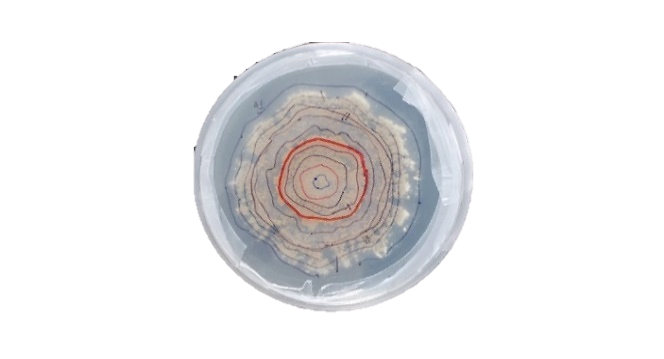

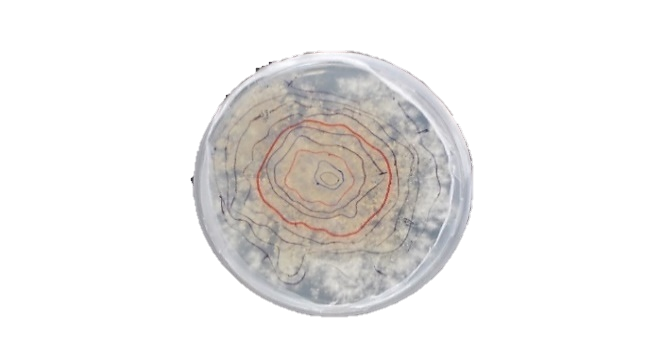


**Appendix S3** Confrontation bioassays between *Escovopsis* morphotypes and fungal cultivars. Letters Y, B1, B2, B3, TB represent five *Escovopsis* morphotypes confronted with CAd. (*Apterostigma dentigerum* cultivar, middle row) and with CAp. (*Apterostigma pilosum* cultivar, bottom row). The top row contains control challenges with no cultivar on the plates.

**
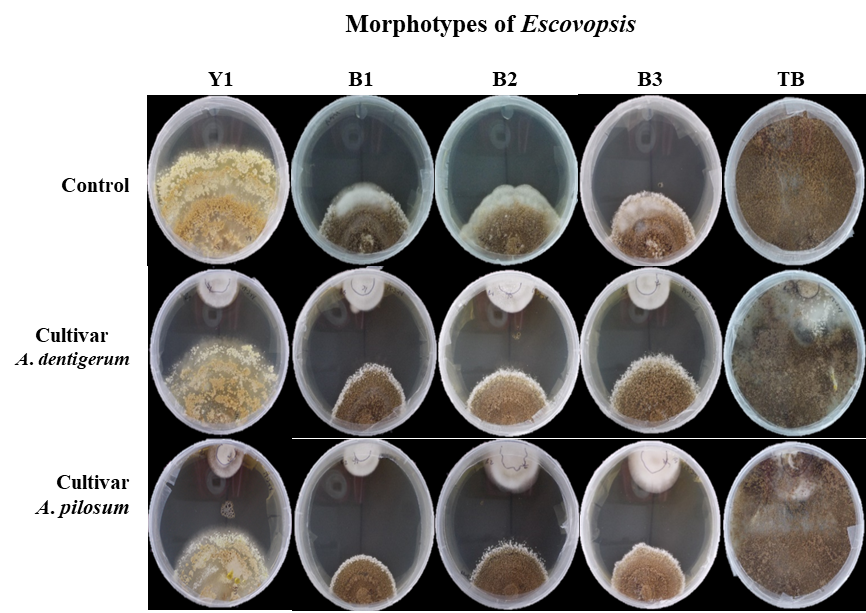
**

**Appendix S4** Cyclic peptides names and structures identified from extracts of five *Escovopsis* morphotypes.

| No. | Mass | Compound name | Structure |
| --- | --- | --- | --- |
| 1 | 115.01 | Cyclo (Gly-Gly) |  |
| 2 | 129.03 | Cyclo (Ala-Gly) |  |
| 3 | 157.03 | Cyclo (Gly-Val) |  |
| 4 | 171.05 | Cyclo (Gly-Leu) |  |
| 5 | 173.04 | Cyclo (Asp-Gly) |  |
| 6 | 185.06 | Cyclo (Leu-Ala) |  |
| 7 | 199.04 | Cyclo (Val-Val) |  |
| 8 | 213.06 | Cyclo (Leu-Val) |  |
| 9 | 245.05 | Cyclo (Phe-Pro) |  |
| 10 | 205.05 | Cyclo (Gly-Phe) |  |
| 11 | 219.01 | Cyclo (Ala-Phe) |  |
| 12 | 221.06 | Cyclo (Tyr-Gly) |  |
| 13 | 231.06 | Cyclo (Asp-Asp) |  |
| 14 | 247.06 | Cyclo (Phe-Val) |  |
| 15 | 258.12 | Cyclo (Ala-Trp) |  |
| 16 | 244.06 | Cyclo (Gly-Trp) |  |

**Appendix S5** Two-way ANOVA of *Escovopsis* growth area in confrontations with different fungal cultivars of *Apterostigma* spp.

| Source | *Df* | *SS* | *MS* | *F* |
| --- | --- | --- | --- | --- |
| Fungal cultivar | 2 | 114.1 | 57.06 | 7.27** |
| *Escovopsis* morphotypes | 4 | 945.0 | 236.26 | 30.14*** |
| *Escovopsis* morphotypes x Fungal cultivar | 8 | 59.3 | 7.41 | 0.945 NS |
| Error | 40 | 313.5 | 7.84 |  |

Note: ** = p < 0.01; *** = p < 0.0001; NS = No significant.

**Appendix S6** Interactions that were statistically significant.

| **Tukey´s multiple comparison** | **Mean Diff.** | **Summary** | **95% CI of diff** | ***P*** |
| --- | --- | --- | --- | --- |
| Y ct vs B1 vs CAd. | 14.37 | *** | 5.541 to 23.20 | < 0.0001 |
| Y ct vs B1 vs CAp. | 12.57 | *** | 3.742 to 21.40 | < 0.0001 |
| Y ct vs B2 vs CAp. | 8.832 | * | 0.00489 to 17.6 | 0.012 |
| Y vs CAd. vs B1 vs CAd. | 11.38 | *** | 3.211 to 19.56 | 0.003 |
| Y vs CAd. vs B1 vs CAp. | 9.585 | ** | 1.412 to 17.76 | 0.001 |
| Y vs CAp. vs B1 vs CAd. | 10.2 | ** | 2.025 to 18.37 | 0.017 |
| Y vs CAp. vs B1 vs CAp. | 8.398 | * | 0.2259 to 16.57 | 0.009 |
| B1 ct vs TB vs CAd. | -8.475 | ** | -17.30 to 0.3527 | 0.019 |
| B1 vs CAd. vs B3 ct | -10.07 | * | -18.90 to -1.243 | 0.040 |
| B1 vs CAd. vs B3 vs CAd. | -10.69 | ** | -18.86 to -2.517 | 0.008 |
| B1 vs CAd. vs TB ct | -16.19 | *** | -24.36 to -8.019 | < 0.0001 |
| B1 vs CAd. vs TB vs CAd. | -16.17 | *** | -24.34 to -8.000 | < 0.0001 |
| B1 vs CAd. vs TB vs CAp. | -15.96 | *** | -24.79 to -7.135 | < 0.0001 |
| B1 vs CAp. vs B3 vs CAd. | -8.891 | * | -17.06 to -0.7183 | 0.004 |
| B1 vs CAp. vs TB ct | -14.39 | *** | -22.57 to -6.220 | < 0.0001 |
| B1 vs CAp. vs TB vs CAd. | -14.37 | *** | -22.55 to -6.201 | < 0.0001 |
| B1 vs CAp. vs TB vs CAp. | -14.16 | *** | -22.99 to -5.336 | < 0.0001 |
| B2 vs CAp. vs TB ct | -10.66 | ** | -18.83 to -2.483 | < 0.0001 |
| B2 vs CAp. vs TB vs CAd. | -10.64 | ** | -18.81 to -2.464 | < 0.0001 |
| B2 vs CAp. vs TB vs CAp. | -10.43 | ** | -19.25 to -1.599 | < 0.0001 |
| B3 vs CAp. vs TB ct | -9.233 | * | -17.41 to -1.061 | 0.005 |
| B3 vs CAp. vs TB vs CAd. | -9.214 | * | -17.39 to -1.041 | 0.002 |
| B3 vs CAp. vs TB vs CAp. | -9.004 | * | -17.83 to -0.1763 | 0.004 |
